# Implementing modular design principles for molecular homogeneous catalyst discovery†

**DOI:** 10.1039/d6cy00980h

**Published:** 2026-10-09

**Authors:** Francis Millward, Benjamin S. Grills, Elizabeth M. Dauncey, Kenneth F. Clark, Benjamin N. Bhawal

**Affiliations:** a School of Chemistry, The University of Edinburgh Edinburgh EH9 3FJ UK ben.bhawal@ed.ac.uk; b Early Chemical Development, Pharmaceutical Sciences, R&D, AstraZeneca Macclesfield SK10 2NA UK

## Abstract

Optimising the structure of molecular homogeneous catalysts is essential to ensure they perform as desired. This can be particularly challenging when seeking to realise selectivity in a catalytic process. The modular design strategy provides a powerful approach for evaluating catalyst structure by generating large and diverse libraries of catalysts. Evaluating these catalyst libraries gives invaluable insights into catalyst function and informs the development of more efficient catalysts. In this perspective, we look at various strategies that have been used to implement modular designs to develop new ligands, organocatalysts and photocatalysts.

## Abstract 2

Modular catalyst design as a strategy to access large and diverse catalyst libraries.

## Graphical abstract

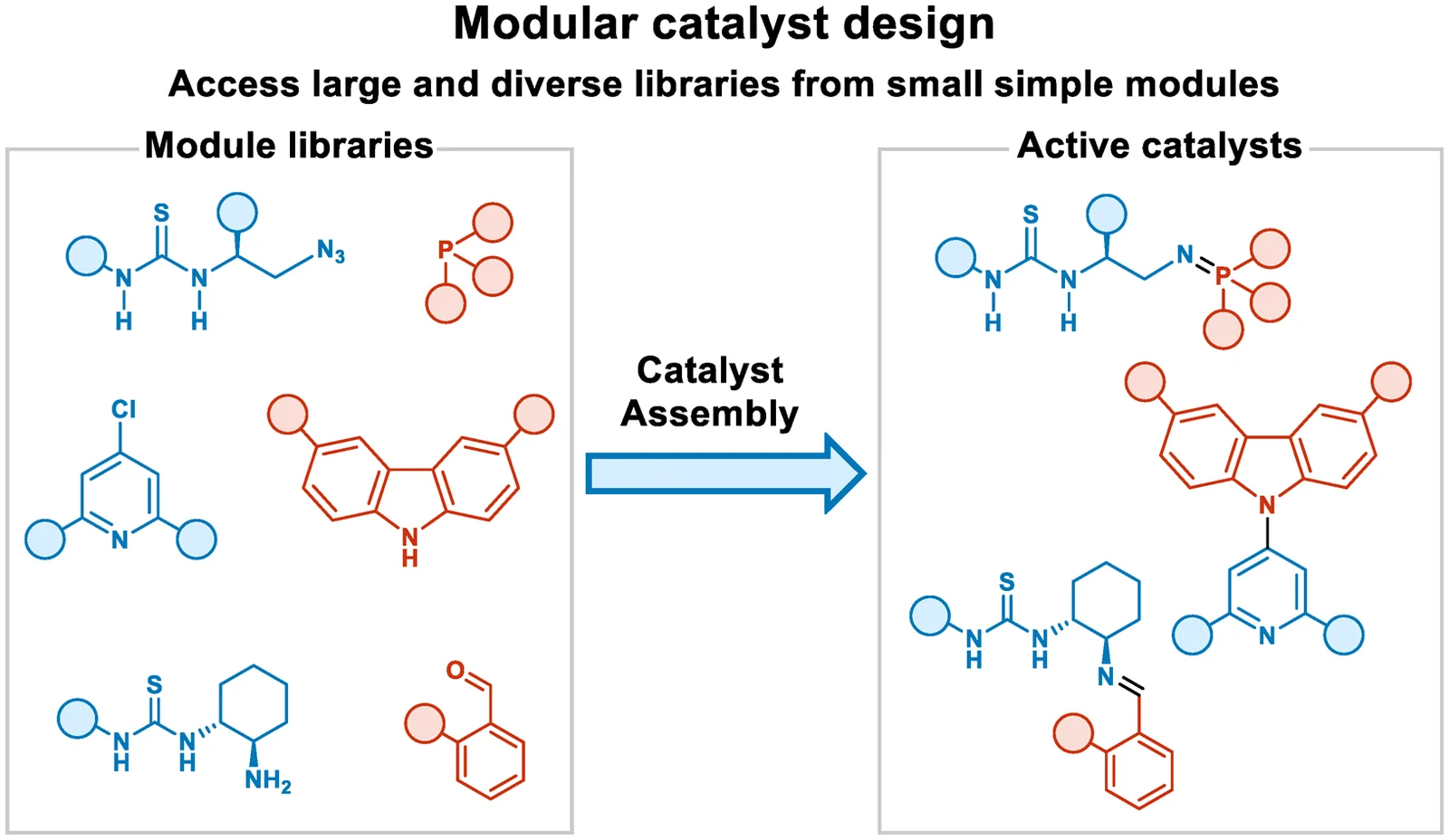

## 1 Introduction

The demand to rapidly access targets characterised by increasing molecular complexity from the pharmaceutical,^1–3^ agrochemical^4^ and other research fields^5^ has provided an impetus for the development of new chemical transformations. Molecular homogeneous catalysis has been pivotal in addressing these demands. This is due to the ability to synthesise ligands and organocatalysts to fine-tune the reactivity and 3-dimensional structure of the catalyst to achieve the desired reaction outcome with regards to chemo-, regio-, stereo- and site-selectivity.^6,7^ Thus, catalyst structure optimisation is essential, and can be guided by a strong understanding of the catalytic mechanism and catalyst function. However, when limited mechanistic insight is available, performing a broad survey of potential catalysts is valuable to rapidly gain this understanding. The implementation of a modular design strategy offers a simple yet effective means for achieving this.

Modular design strategy involves breaking down a complex system into smaller, relatively simple modules (Scheme 1). When applied in catalysis, the different modules tend to confer a specific function to the catalyst, such as a binding site for a transition metal, a hydrogen bond donor for binding to a substrate or can even simply act as a chiral backbone to induce a productive 3D structure for the catalyst. This design strategy enables the construction of modular catalyst libraries, where each type of module can be prepared separately, and then any member of one module class can be combined with any member of the other module class, generating a 2D library of active catalysts. Ideally, the assembly of the modules should be performed using robust synthetic transformations that are tolerant to any functionality present within the modules. If possible, *in situ* module assembly circumvents the need to isolate each member of the catalyst library and instead allows direct evaluation of the catalyst's performance in the transformation of interest.

**Scheme 1:**
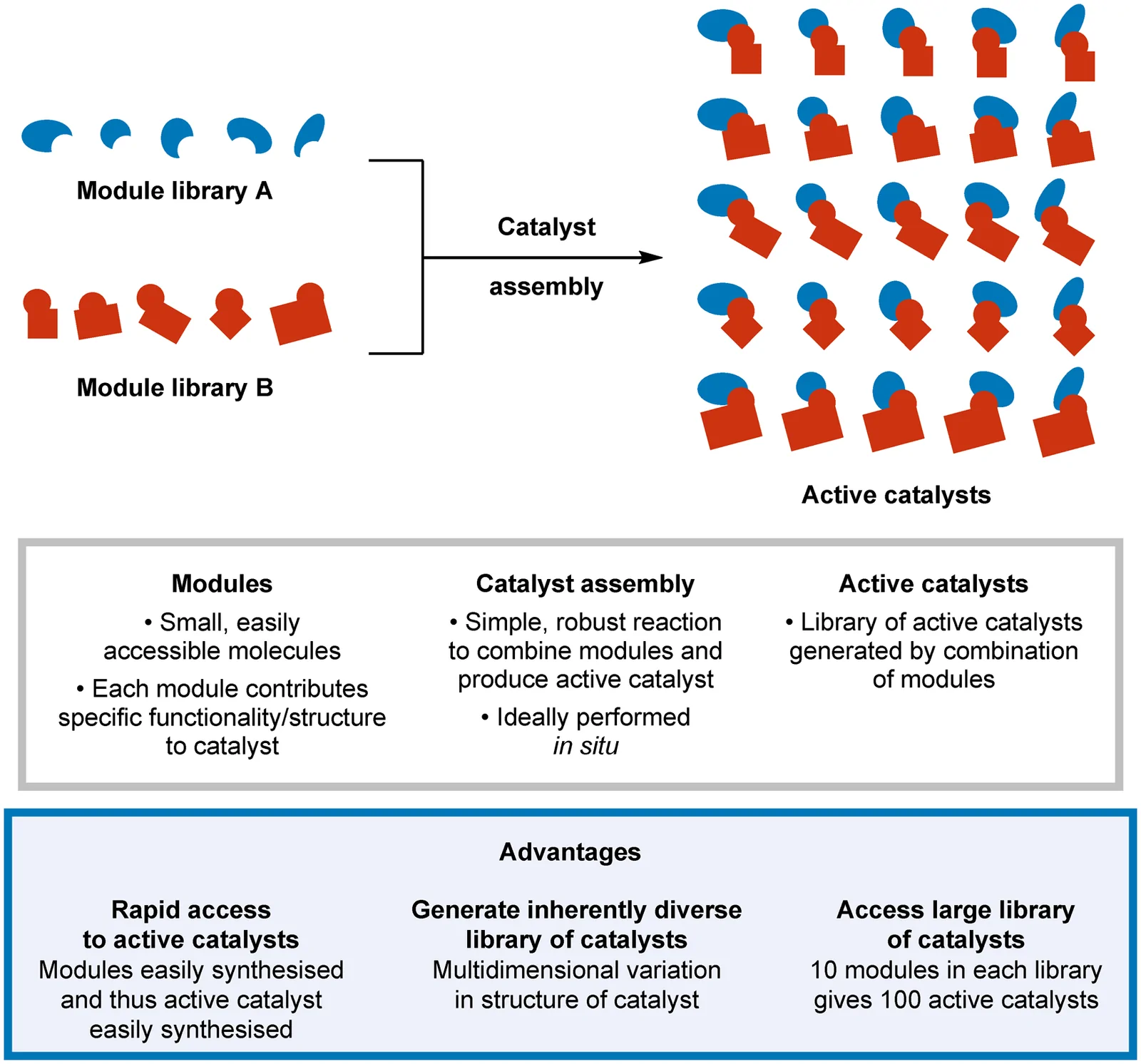
Modular catalyst design.

Adopting a modular design strategy in the development of new catalysts offers numerous advantages. In particular, it facilitates rapid access to a large and diverse library of catalysts. For a bimodular design, 10 modules in each library would give a library of 100 active catalysts. As both the modules can be varied, this leads to the generation of a more inherently diverse library of catalysts. This is particularly valuable because structural features that govern catalytic performance can only be understood by studying a wide range of catalysts, including less efficient ones. With the advent of machine learning, large and diverse datasets are essential to provide the broadest picture and avoid any potential human bias^8,9^ to derive the most accurate conclusions. It should be noted, though, that the implementation of a modular catalyst design does introduce its own biases and the design will restrict the selection of catalysts evaluated.

In this perspective, we explore different approaches for implementing modular design principles for catalyst discovery, with selected case studies chosen to illustrate how this has solved challenges in small molecule synthesis with a focus on the design of novel ligands, organocatalysts and photocatalysts. We have prioritised examples that use simple approaches for the assembly of the modules and enable direct access to the active catalysts without the requirement for any further synthetic modification. The majority of this review focusses on bimodular catalysts which we have categorised according to the mode of assembly: covalent assembly, non-covalent assembly, and dynamic assembly, with a final section that discusses two-dimensional metal–ligand libraries. Enzyme catalysis is arguably the quintessential example of modular catalyst design,^10–13^ wherein different amino acid modules are linearly assembled to give the active catalyst and has inspired the design of other combinatorial catalysts and ligands.^14–17^ However, as these have been covered extensively elsewhere, this will not be included in this perspective.

## 2 Implementing modular design

Ideally, when using a modular design strategy, one wants to access as large and diverse a library of catalysts in as few synthetic steps as possible. In particular, performing assembly of the catalyst as late as possible in the synthetic sequence significantly reduces the number of synthetic steps required to produce the catalyst library.

To illustrate this, we present three simplified scenarios (Scheme 2). In scenario A, the catalysts are synthesised through simple linear sequences. In scenario B, bimodular catalysts are prepared by assembling the catalysts in the final step of the synthetic sequence. In scenario C, the catalysts are assembled at an early stage in the synthesis. In all three scenarios, the longest linear sequence to prepare the active catalysts is 4 steps. These are arbitrary examples to demonstrate the advantages of implementing a modular design strategy and how to maximise the efficiency of this approach.

**Scheme 2:**
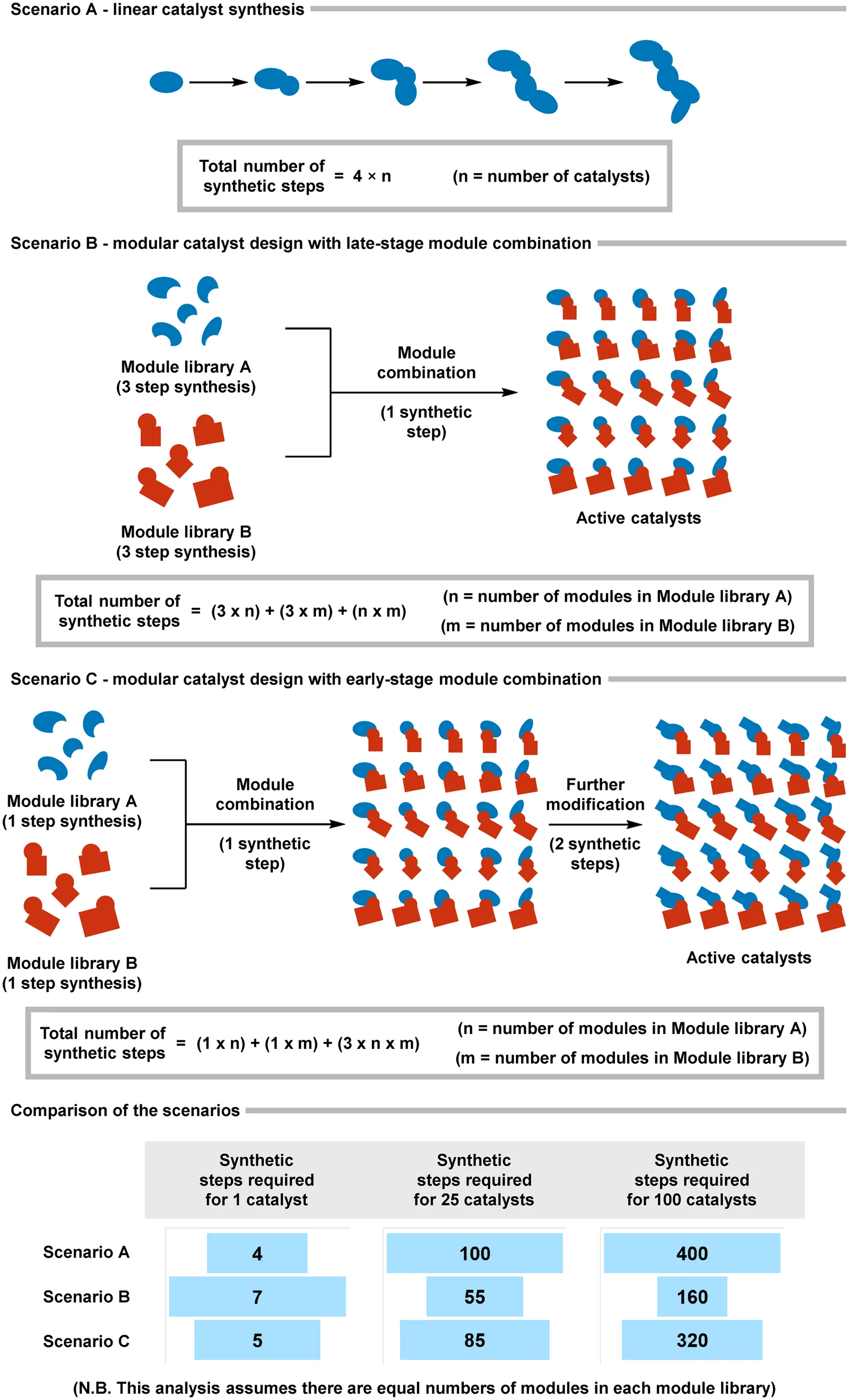
Best practice in implementing modular catalyst design.

For scenario A, while it only takes 4 steps to make the first catalyst, an additional 4 synthetic steps are required to make each iteration of the catalyst. Thus, a library of 25 catalysts would require 100 synthetic steps. For scenario B, 7 synthetic steps are required to prepare a single catalyst. However, this strategy benefits from “returns to scale”; a library of 25 catalysts can be prepared in just 55 synthetic steps, almost half the number required in scenario A. For scenario C, the first catalyst is accessed in just 5 steps, but a library of 25 catalysts would require a total of 85 steps, marginally fewer steps than involved in scenario A. Thus, when planning a modular catalyst design, it is beneficial to prioritise disconnecting the catalyst so that the modules are assembled as late as possible to ensure maximum efficiency.

## 3 Covalent assembly of bimodular catalysts

The simplest approach to implement a modular design strategy is to disconnect the catalyst into two distinct modules which can be combined using a simple synthetic transformation. Various disconnections can be envisaged, but from a practical perspective the assembly method should rely on easily accessible functional groups and require limited purification or, ideally, be performed *in situ*.

An early example of modular catalyst design was reported by the Trost group.^18^ Asymmetric allylic alkylation had recently emerged as a powerful transformation for the functionalisation of simple allylic starting materials by reaction with a variety of nucleophiles.^19–21^ Achieving enantioselective processes initially proved challenging, with classic chiral phosphine ligands (*e.g.*, BINAP, DIOP) providing unsatisfactory results with Pd-catalysis. To address this challenge, the Trost group opted to prepare and evaluate a range of new chiral phosphine ligands (Scheme 3). This was executed by combining 2-diphenylphosphinobenzoic acid (**Module 1A**) with a range of chiral alcohols and amines (**Module 1B**) through simple Steglich esterification or amide coupling. The wide availability of suitable chiral amines and alcohols enabled evaluation of a diverse range of catalysts, including mono-, bi- and tridentate ligands. Thus, they were able to identify a chiral phosphine ligand capable of promoting their model reaction, the desymmetrisation of a *meso*-2-ene-1,4-diol, in high enantioselectivity. Initially, monoalcohols, diols and triols were explored, which suggested *C*_2_ symmetric bidentate ligands to be most effective. Rigidification of the ligand structure by transitioning from esters to amides led to the identification of 1,2-diphenylethanediamine as the optimal chiral backbone. In addition to the intramolecular process, this ligand also enabled the asymmetric intermolecular allylic alkylation of both carbon and nitrogen nucleophiles. Further refinement of this ligand led to the development of the Trost ligand,^22^ which is now the standard ligand used in Pd-catalysed asymmetric allylic alkylations, working with a range of allylic alcohols and nucleophiles.^23^ Although only one module was varied in this work, it should be noted that the wide availability of both carboxylic acids and alcohols or amines makes this strategy for modular catalyst design both conceptually and practically simple to implement.

**Scheme 3:**
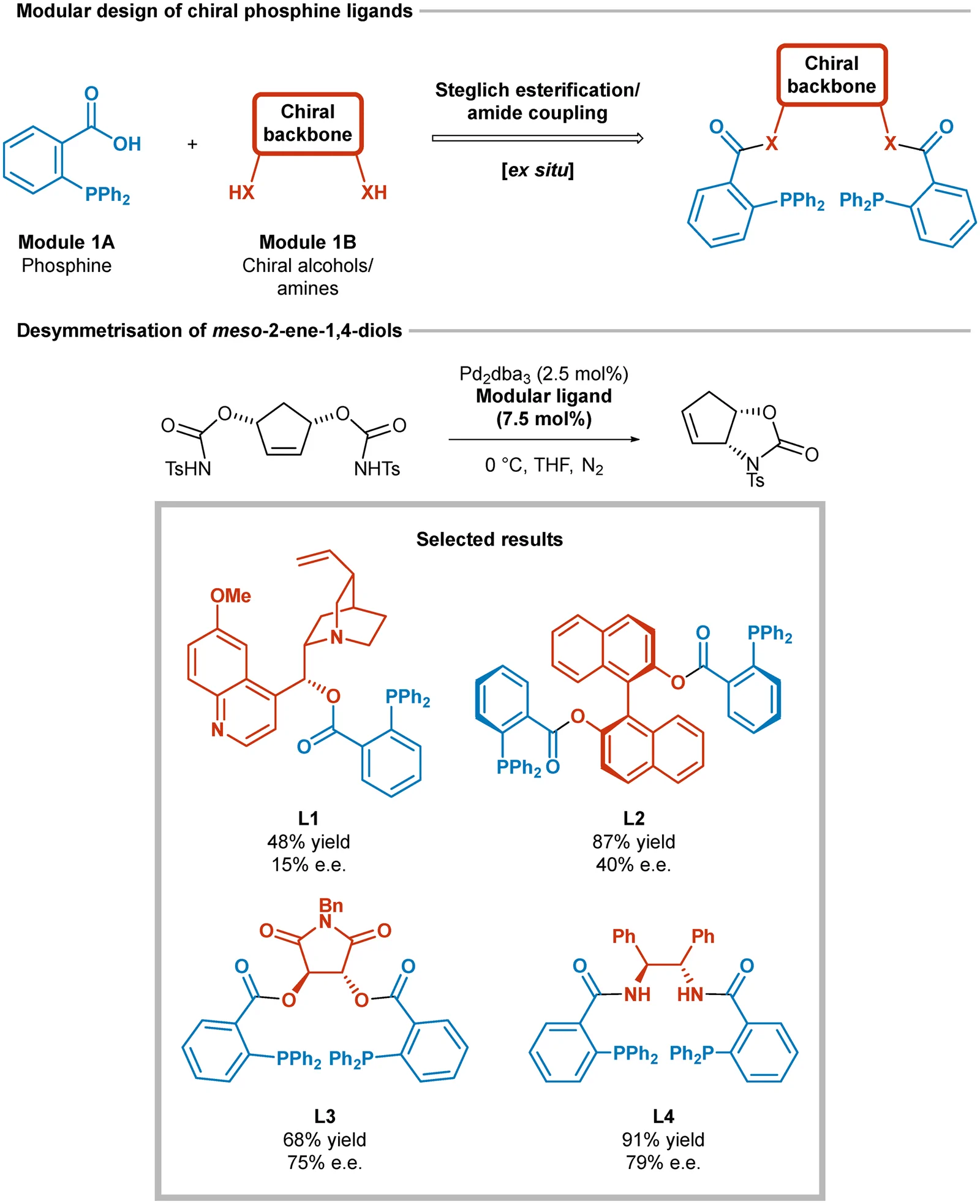
Modular catalyst design in the development of chiral phosphine ligands for Pd-catalysed asymmetric allylic alkylation as reported by Trost and co-workers.^18^

In 2013, the Dixon group reported bifunctional iminophosphoranes (BIMPs) as a new class of superbase organocatalysts (Scheme 4). Key structural features of these catalysts include a hydrogen-bond donor motif, such as a thiourea or amide, and the highly basic iminophosphorane functional group.^24,25^ The catalysts are assembled through the Staudinger reaction of a chiral azide (**Module 2A**) and a phosphine (**Module 2B**). Both of these modules can be varied with the basicity of the iminophosphorane functional group dictated by the phosphine and the chiral azide module modulating the steric environment of the catalyst and the strength of the hydrogen bond donor. Notably, the chiral azide module can be further disconnected into a hydrogen bond donor module and chiral backbone module, thus providing three points of variation in the overall structure of the BIMP catalysts. Due to the clean assembly of the BIMP catalyst through the Staudinger reaction, with only N_2_ produced as a by-product, these catalysts can be synthesised *in situ*, making them ideal for modular synthesis.

**Scheme 4:**
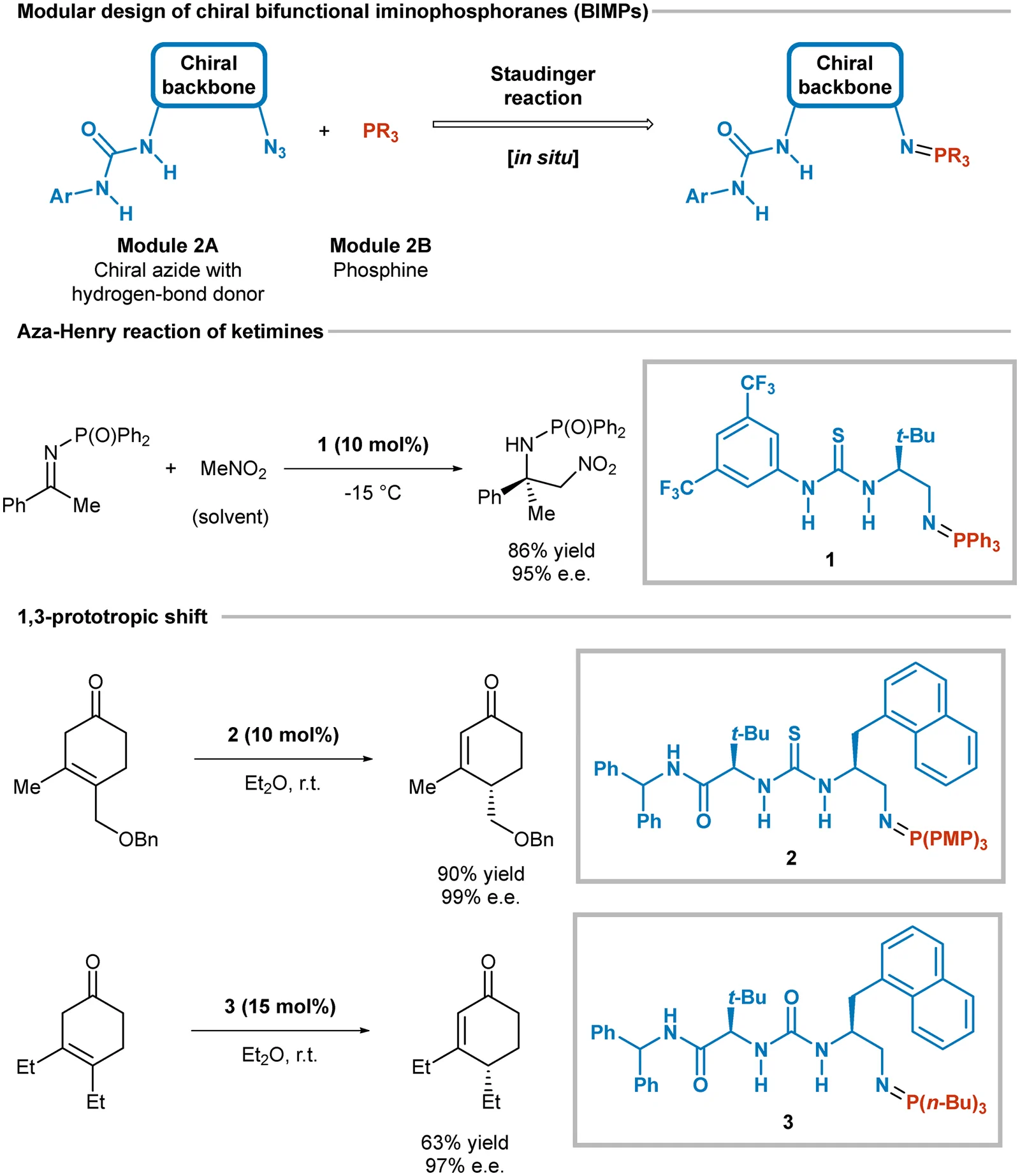
Design of modular bifunctional iminophosphoranes and their application as superbase organocatalysts as reported by Dixon and co-workers.^24,25,31^

Since their inception, these superbase organocatalysts have enabled a range of highly challenging asymmetric transformations, including the aza-Henry reaction of ketimines,^25^ and 1,3-prototropic shifts^31^ amongst others.^26–30^ For the 1,3-prototropic shift, the ability to modify both the phosphine and chiral azide modules proved advantageous; fine-tuning of the chiral backbone afforded high enantioselectivities and, with more challenging substrates, a trialkylphosphine module was used to increase the Brønsted basicity of the catalyst and achieve sufficient reactivity.

Inspired by the seminal report from the Cramer group detailing a chiral cyclopentadiene–Rh catalyst for enantioselective C–H functionalisation,^32^ a range of new chiral cyclopentadiene ligands have been reported.^33^ Translating these advances to achieve more sustainable transformations using Co-based catalysts presented a challenge due to the limited stability of chiral disubstituted cyclopentadiene Co complexes, leading to low catalytic turnovers. Using pentasubstituted chiral cyclopentadiene (Cp^V^) ligands confers greater stability to the Co-complexes, but development of these ligands is hampered by their challenging synthesis. To address these issues, Cramer and co-workers designed a new route to chiral Cp^V^ ligands (Scheme 5) relying on the dialkylation of 1,2,3-trisubstituted cyclopentadienes (**Module 3B**)^34^ with chiral dibromides (**Module 3A**).^35^ Variation of both reaction partners led to the preparation of over 30 new chiral cyclopentadienes with their complexation with Co, Rh and Ir performed directly following dialkylation without intermediate purification of the cyclopentadiene. The application of these catalysts was demonstrated in various transformations including the Co-catalysed (3 + 2) C–H spiroannulation of an *N*-sulfonyl ketimine, illustrating the improved stability of the cyclopentadiene–Co complex and subsequent enhanced reactivity. In the Rh-catalysed allylic C–H amination, tuning of both the chiral backbone and substituents on the cyclopentadiene ligand proved pivotal to achieve high enantioselectivities.

**Scheme 5:**
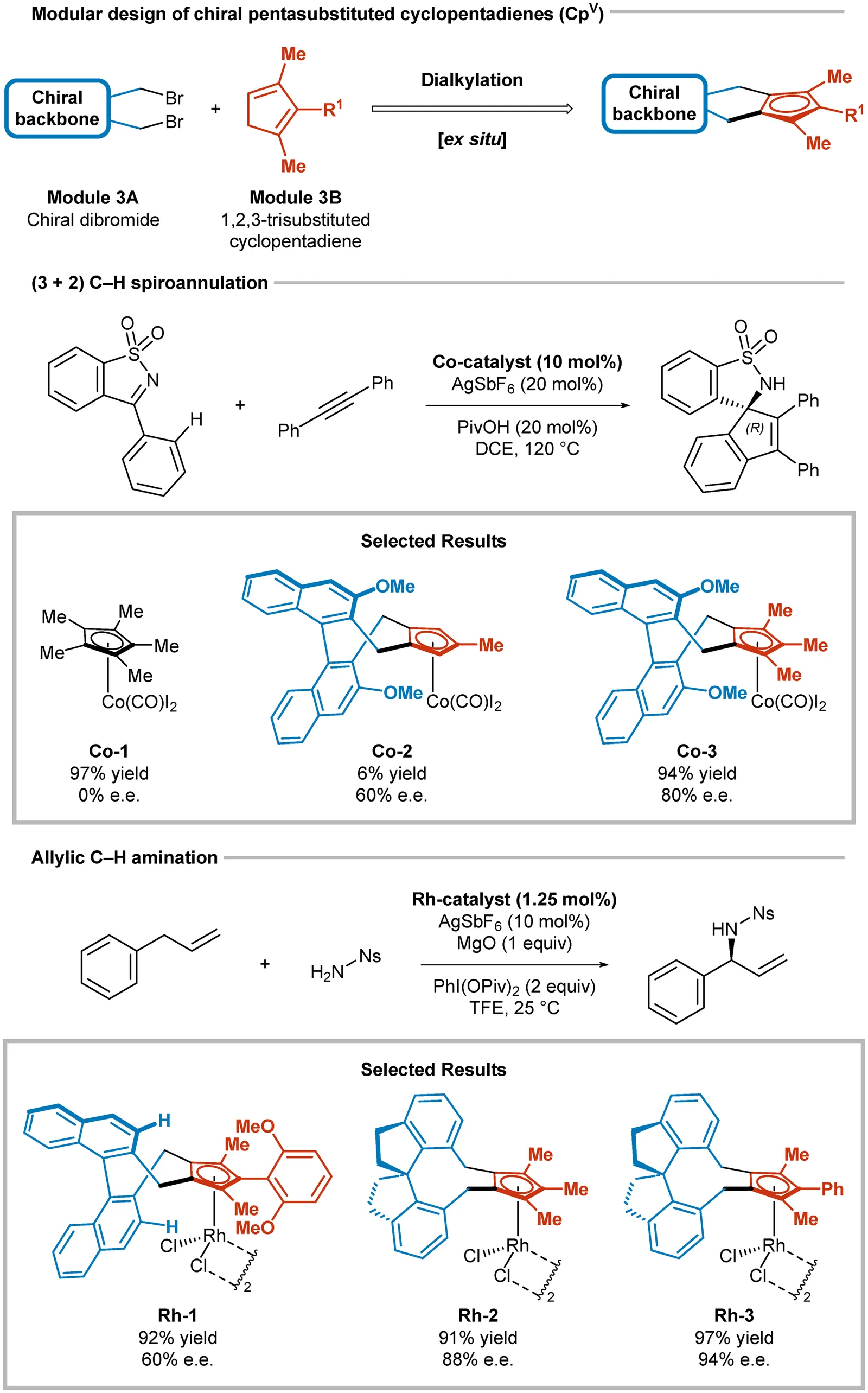
Design of modular chiral cyclopentadiene ligands and application in enantioselective C–H functionalisation as reported by Cramer and co-workers.^35^

Organic dye photocatalysts are considered as more sustainable alternatives to transition metal photocatalysts featuring ruthenium(ii) and iridium(iii) centres.^36,37^ Historically the key advantage of using transition metal catalysts was their tuneable optoelectronic properties driven by well-designed ligand modifications. Many of the organic photocatalyst designs originally used for light-driven reactions, such as those featuring diphenylketone, acridine, or xanthene motifs, lacked the ability to tune the photochemical properties of these catalysts. However, the advent of donor–acceptor photocatalyst designs, archetypically illustrated by the cyanobenzene class of photocatalysts, allows modulation of the photophysical properties of these catalysts by variation of the donor and acceptor modules.^38^

Cyanobenzene photocatalysts can often be assembled by simple S_N_Ar reactions between the donor and acceptor modules, providing a streamlined route to access a diverse range of photocatalysts. This modular tunability was explored by the Zeitler group (Scheme 6), who surveyed the impact of varying both the donor (**Module 4A**) and acceptor (**Module 4B**) modules.^39^ Systematic evaluation of their photocatalysts showed that this design principle provided access to a wide range of ground state oxidation potentials (+1.79 V to +1.02 V) and ground state reduction potentials (−1.92 V to −1.16 V). The utility of these catalysts was demonstrated through various transformations, including decarboxylative Giese addition and reductive C–O cleavage with subsequent pinacol coupling.

**Scheme 6:**
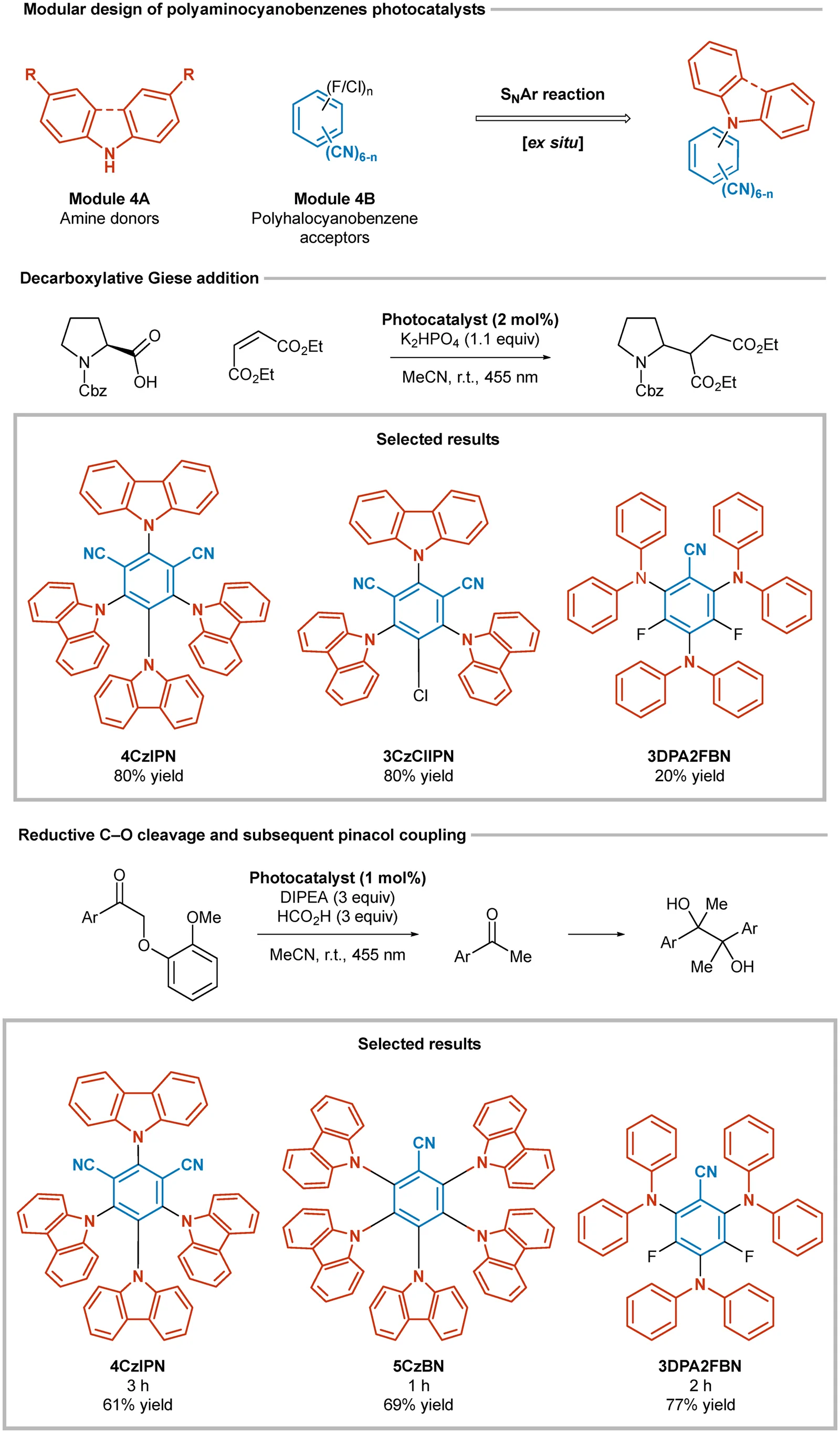
Modular design of organophotoredox catalysts and application as reported by Zeitler and co-workers.^39^

Later, the groups of König, Zhu and Rueping also exploited the modularity of cyanobenzene organic dyes to target red-light photoredox catalysts that benefit from the superior light penetration and improved functional group tolerance of longer wavelength light sources.^40^

More recently, the Yoon and Zysman-Colman groups expanded on this strategy to develop chiral organic photocatalysts for enantioselective photocatalytic transformations (Scheme 7).^41^ They amalgamated the structural features of these photocatalysts with pyridine-2,6-bisoxazoline (PyBOX) ligands, a well-established group of privileged chiral ligands for enantioselective catalysis. These catalysts can be split into two modules; 1) the PyBOX acceptor (**Module 5A**) which contains the chiral elements required to confer enantioselectivity and 2) a carbazole donor moiety (**Module 5B**) that can be varied to tune the photophysical properties of the catalyst. Having these two handles to tune the catalysts proved critical in the development of a trio of enantioselective photocatalytic processes, with the ability to modify both the photophysical properties and the chiral pocket of the catalyst proving essential to achieve high selectivity in all three cases.

**Scheme 7:**
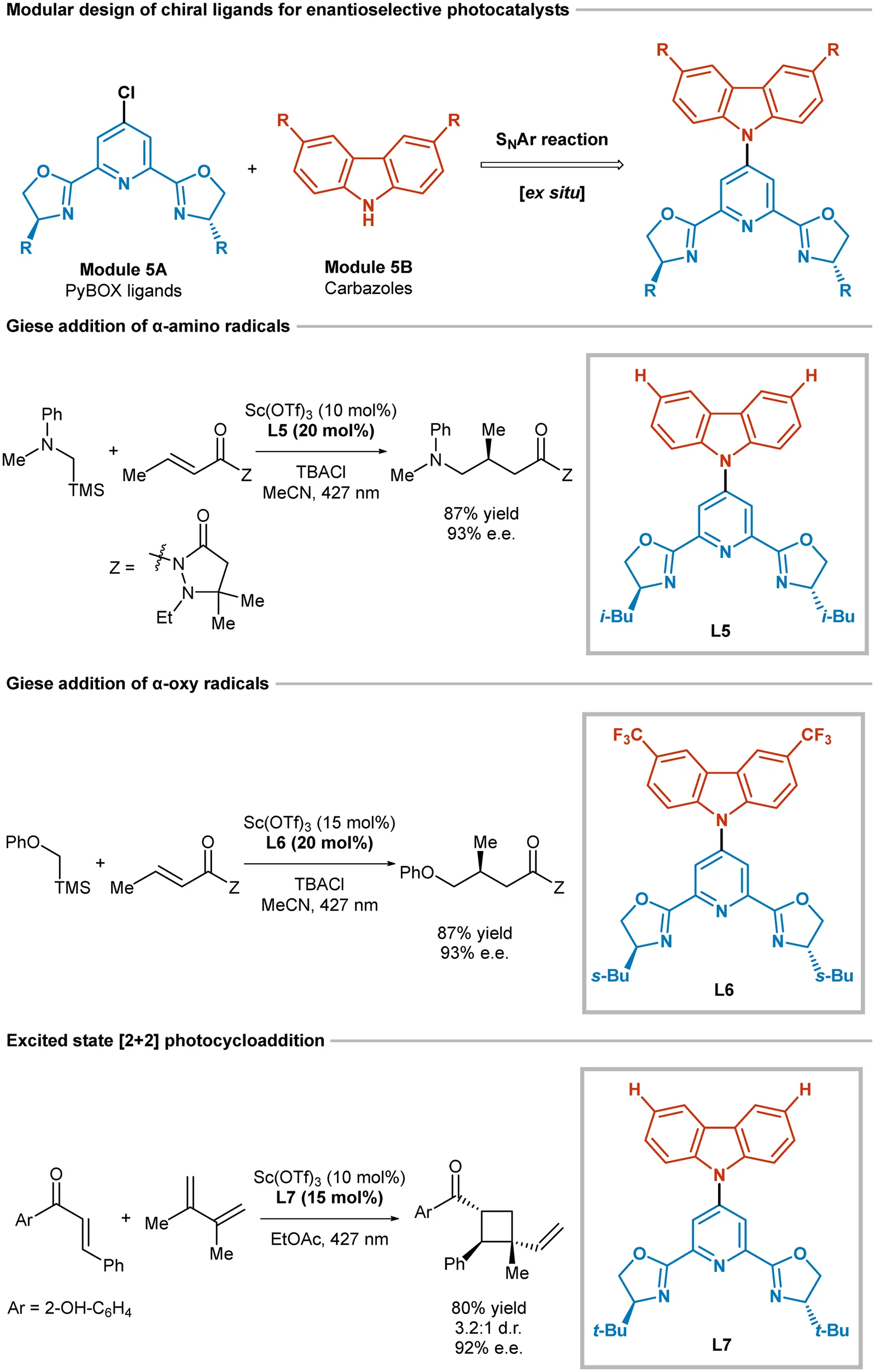
Modular design of photoredox active chiral ligands for enantioselective photoredox catalysis as reported by the Yoon and Zysman-Colman groups.^41^

## 4 Supramolecular assembly of bimodular catalysts through non-covalent interactions

Supramolecular concepts exploiting strong non-covalent interactions can be used to assemble modular catalysts by *in situ* combination of suitable modules.^42–44^ This requires judicious design of the modules to ensure compatibility between the interactions to assemble the catalyst, and interactions between substrates to afford the desired reactivity. Furthermore, complementary binding motifs between the modules are required to achieve asymmetric assembly between two different modules. Thus, it can be challenging to control these different interactions to give the desired catalyst speciation and recognition with the substrate.

Early examples involved homodimerisation to give bidentate ligands for transition metal catalysis,^45^ and, in 2004, the Reek group were first able to implement a modular design strategy using *in situ* self-assembly of non-identical modules for Pd-catalysed allylic alkylation (Scheme 8).^46^ This was achieved by using the non-covalent interaction between Zn-porphyrins and pyridines as a complementary binding motif. The Zn-porphyrin modules (**Module 6A**) possessed phosphite substituents, while the pyridine was embedded in either phosphine or phosphite ligands (**Module 6B**). Using this design strategy, the authors were able to evaluate a library of 48 phosphite–phosphine and phosphite–phosphite bidentate ligands from just 14 monodentate modules. Unexpectedly, in the asymmetric allylic alkylation of 1,3-diphenylallyl acetate with dimethyl malonate, they found one of their Zn-porphyrin phosphite modules gave high enantioselectivity on its own, albeit with limited conversion. By contrast, their *in situ* assembled bidentate ligands were more active but gave moderate enantioselectivities, with a switch in the preferred enantiomer formed occasionally observed depending on the combination of modules used. This report was the first example of using non-covalent interactions to assemble a bimodular catalyst, demonstrating this as a viable strategy. However, it also illustrates some of the potential drawbacks; namely the requirement for more complex functionality such as a porphyrin to achieve the desired molecular recognition between the modules.

**Scheme 8:**
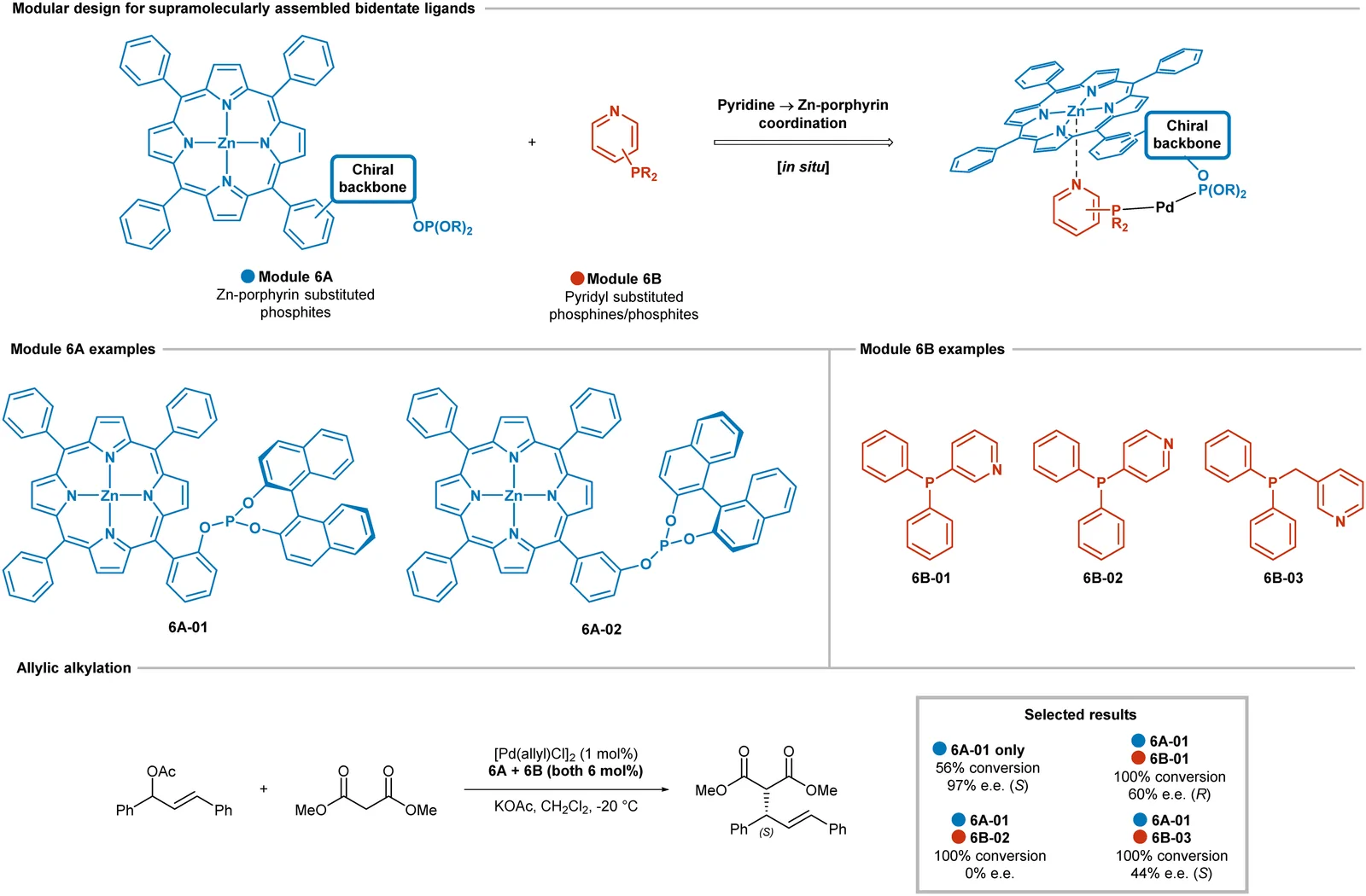
Modular design and application of bidentate ligand assembled through pyridine to zinc porphyrin coordination as reported by Reek and co-workers.^46^

Later, the group of Breit took inspiration from DNA base pairs to formulate an approach for modular self-assembled catalysts (Scheme 9).^47^ They prepared libraries of 2-aminopyridyl phosphines (**Module 7A**) and phosphinoisoquinolones (**Module 7B**) that bind together through a hydrogen-bonding network in a manner akin to that of adenine–thymine base pairs. They evaluated a 4 × 4 matrix of these ligands in the Rh-catalysed hydroformylation of 1-octene and identified a module combination that gave a TOF of 8643 h^−1^ and 96 : 4 regioselectivity in favour of the linear isomer. Later, the Breit group developed a catalyst evaluation protocol that facilitated the identification of 3 highly selective catalysts from a library of 120 catalysts in just 17 reactions.^48^

**Scheme 9:**
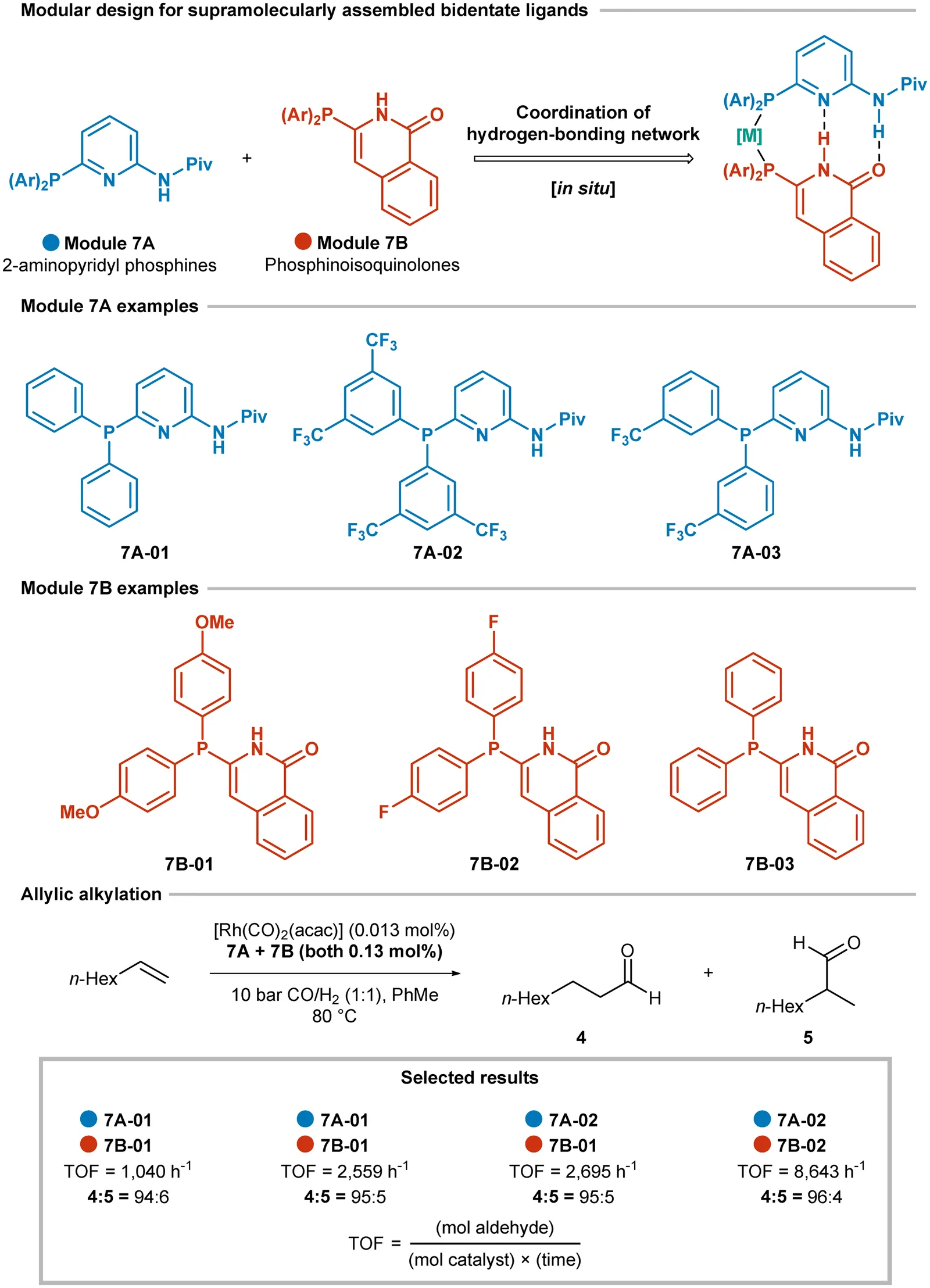
Modular design and application of bidentate ligand assembled through a hydrogen-bonding network as reported by Breit and co-workers.^47^

These concepts have also been extended to the design of modular organocatalysts by the Clarke group (Scheme 10).^49^ This was achieved by modification of both proline and a dipeptide (Pro-Val) through amide coupling with an aminonaphthyridine to give a library of amidonaphthyridine modules (**Module 8A**). To complement this library, they built a second library of achiral aminopyridinones (**Module 8B**) that bind *in situ* through a selective hydrogen-bonding network to the amidonaphthyridine. While the amidonaphthyridines modules can act as a chiral catalyst for the nitro-Michael reaction, low yields and enantioselectivities were observed. Addition of the aminopyridinone module gave catalysts that exhibited both improved activity and enantioselectivity, demonstrating that even achiral modules can modulate the chiral properties of a catalyst. Following this seminal work, numerous other self-assembling organocatalysts have been elaborated.^50–52^

**Scheme 10:**
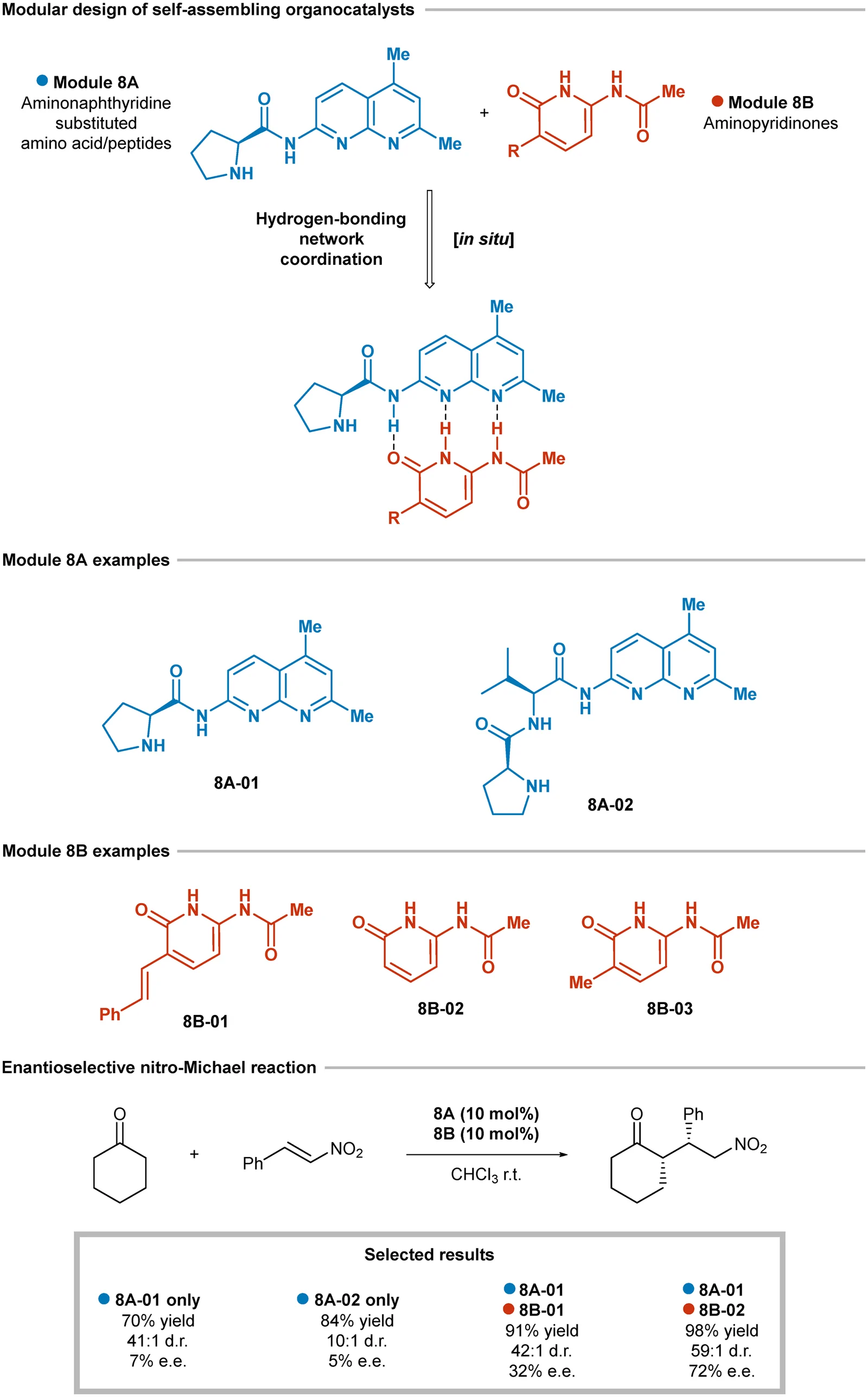
Design of modular organocatalysts and their application in the enantioselective nitro-Michael reaction as reported by Clarke and co-workers.^49^

Since its inception, the chiral counterion strategy has proved a powerful approach to render transition metal catalysed transformations enantioselective.^53–55^ This strategy involves pairing transition metal complexes with chiral counterions and inherently lends itself to the construction of modular catalyst libraries. A seminal demonstration of this was reported by the Ooi group who had developed ammonium phosphine ligands that are paired with chiral BINOL derived anions for Pd-catalysed enantioselective allylation.^56^ Later, they extended upon this approach (Scheme 11) and explored the pairing of chiral ammonium phosphine ligands (**Module 9A**) with chiral phosphates generated *in situ* by deprotonation of the corresponding phosphoric acid (**Module 9B**) to achieve the challenging asymmetric allylic alkylation of a benzothiophenone to form a crowded quaternary centre.^57^ With two chiral species, it is essential to find matched pairs of enantiomers and thus both the *R*- and *S*-enantiomers of the chiral ammonium phosphine ligands were evaluated. In total, 12 ammonium phosphines and 12 phosphoric acids were surveyed for the enantioselective allylation of benzothiophenones. Although this gives a total of 144 ion-pairs, a combinatorial screening strategy was demonstrated that allowed identification of the most efficient pair in just 16 experiments.

**Scheme 11:**
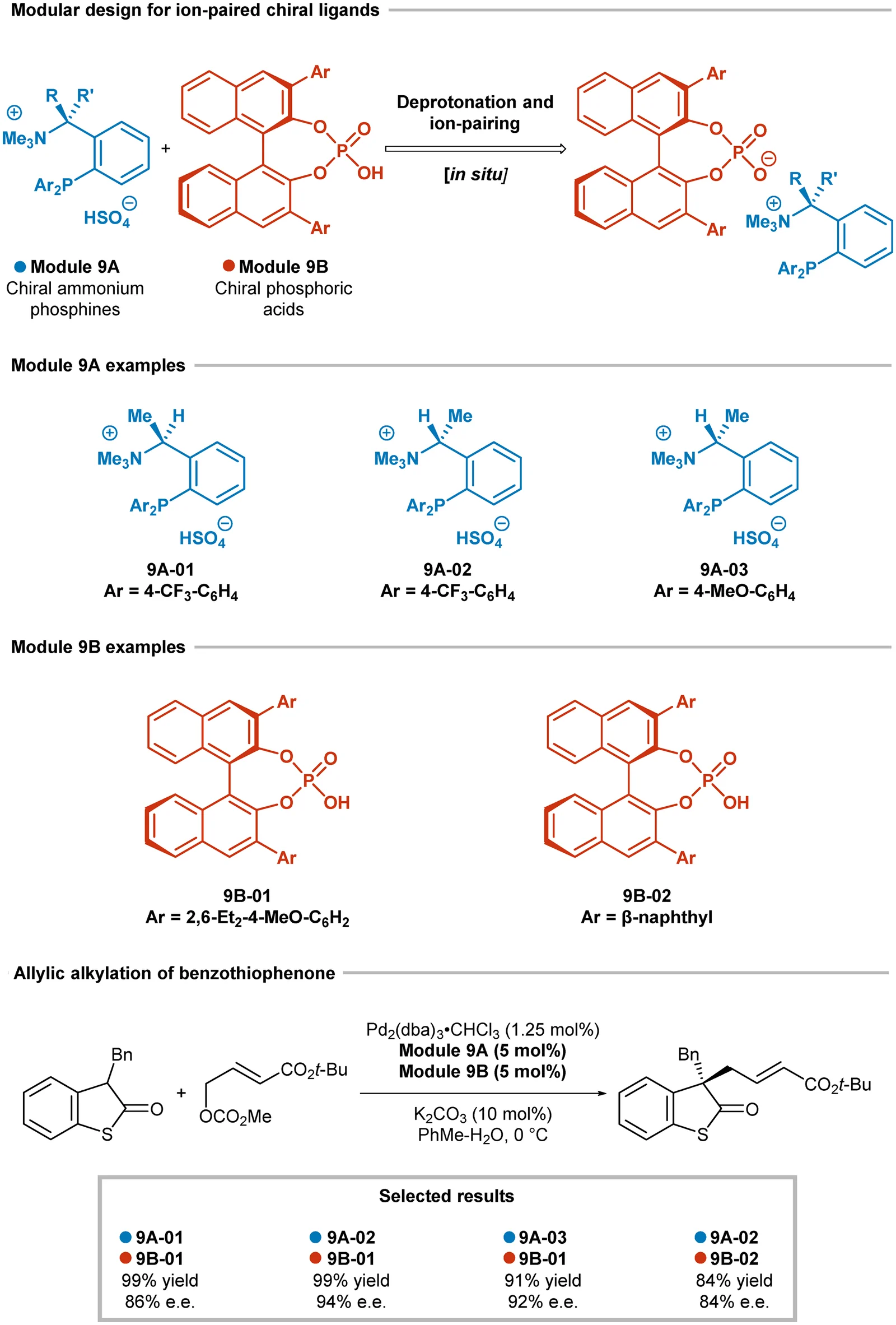
Design of modular ion-pairs and their application in the enantioselective allylic alkylation as reported by Ooi and co-workers.^57^

Later, the Phipps group also explored ion-pairing interactions to assemble supramolecular catalysts, pairing chiral cations with achiral anionic transition metal complexes.^58^ A notable contribution demonstrated the utility of this approach in the development of an enantioselective intermolecular C–H amination (Scheme 12).^59^ The reaction is promoted by an achiral Rh-dimer with a pendant sulfonate group (**Module 10B**) that is paired with a chiral ammonium cation (**Module 10A**). Both of these modules are relatively facile to access, although they are paired through a cation exchange process that is performed *ex situ*. Variation of both modules influenced the yields and enantioselectivities observed in their model reaction.

**Scheme 12:**
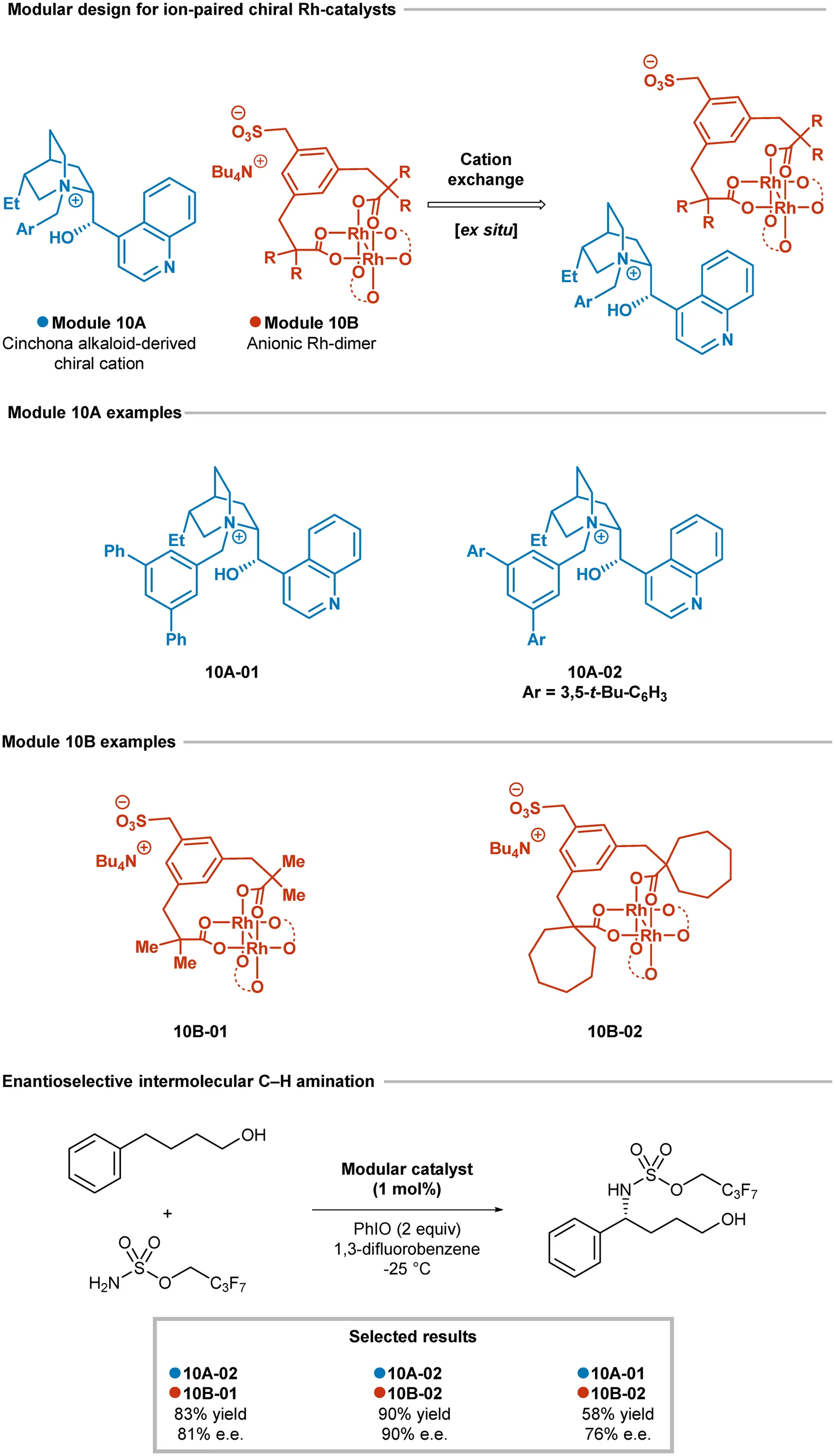
Design of modular ion-paired Rh-dimer catalysts for enantioselective intermolecular C–H amination as reported by Phipps and co-workers.^59^

Recently, the group of Zuccarello reported a photocatalysed deracemization of 2-aryl pyrrolidines facilitated by hydrogen atom transfer (HAT) catalysis (Scheme 13).^60^ Their design relies on a network of complementary non-covalent interactions between both of their modules and the substrate. A chiral phosphoric acid module (**Module 11A**) is deprotonated to generate a potassium phosphate *in situ* and acts as a template to bind to both the substrate through the Brønsted basic site of the phosphate group and 2-pyridylthiol HAT catalysts (**Module 11B**) by coordination between the pyridine and potassium cation. Crucially, the chiral phosphoric acids provide the chiral environment enabling the use of simple achiral thiols to be used as the HAT module, rather than relying on less accessible chiral thiol catalysts. A library of 6 chiral phosphoric acids and 8 achiral thiols were explored by one-factor-at-a-time optimisation of the module pairs, first identifying the optimal chiral phosphoric acid module, and subsequently screening this against the HAT modules. The identity of each module heavily impacted the enantioselectivity of the process and control reactions demonstrated the importance of the pyridyl group in the HAT module in achieving deracemization. While the authors used a stepwise one-factor-at-a-time optimisation to explore different module combinations, future iterations of this approach lend themselves favourably towards a 2D screening mediated by HTE; the commercial availability of the thiol HAT modules allows for an extensive library to be generated with minimal synthetic input. Although there are challenges in controlling a network of non-covalent interactions to avoid unintended interactions, this work provides a powerful exemplar to demonstrate the potential of this approach.

**Scheme 13:**
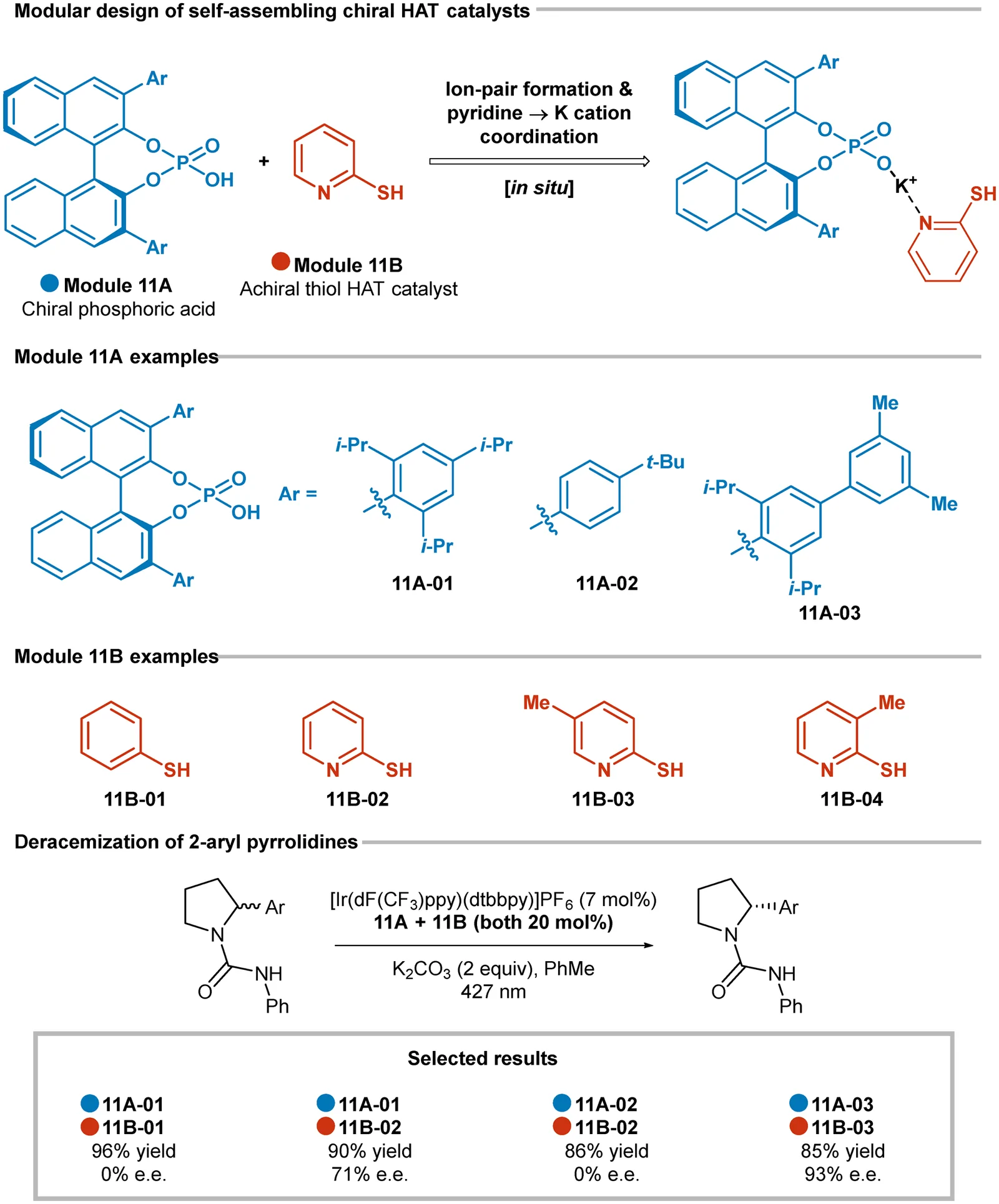
Design of modular HAT catalysts for the deracemization of 2-arylpyrroldines as reported by Zuccarello and co-workers.^60^

## 5 Dynamic catalyst assembly

In dynamic combinatorial chemistry, a library of compounds are assembled in a single reaction vessel from simple building blocks using reversible reactions. As a result, an equilibrium is established and interconversion between the library members occurs leading to a dynamic library of products.^61^ Typical reversible processes used to achieve this include transesterification, imine exchange and thiol–disulfide exchange. In catalyst discovery, this concept holds the potential to allow the simultaneous screening of large libraries of catalysts, offering an alternative strategy to implementing modular catalyst design, wherein a library of catalysts can be accessed *in situ* by reversible assembly of multiple modules. However, as this will lead to the formation of multiple catalyst species in a single reaction vessel, it can be challenging to determine the concentration of each catalyst, particularly as unanticipated interactions or solvent effects may make formation of one catalyst more or less favourable. Thus, it is inherently more challenging to understand the performance of individual catalysts using this approach.

The group of Reetz used this approach to examine the effect of having mixtures of monodentate chiral ligands in transition metal catalysed asymmetric hydrogenation.^62^ At the time, pioneering work by the groups of Orpen and Pringle,^63^ Reetz,^64^ and Feringa^65^ had demonstrated the effectiveness of monodentate phosphonites, phosphites and phosphoramidites as chiral ligands for asymmetric hydrogenation, challenging the dogma that bidentate ligands were required to realise high stereoselectivity.^66,67^ Most of these ligands were used in a 2 : 1 ratio with respect to the transition metal. The group of Reetz later explored the effect of having two different chiral monodentate ligands (Scheme 14, **L**^**a**^ and **L**^**b**^). Rapid ligand exchange means that three different catalytic species exist; two homoligated complexes (**ML**^**a**^**L**^**a**^ and **ML**^**b**^**L**^**b**^) and one heteroligated complex (**ML**^**a**^**L**^**b**^). Despite having this mixture, they theorised that if the catalyst with a heterocombination of ligands is both more active and selective than those with a homocombination, this would still lead to improved enantioselectivities. To examine this hypothesis, the Rh-catalysed hydrogenation of various substrates was examined and, in all cases, the highest enantioselectivities were achieved when using a mixture of ligands. This effect was particularly pronounced in the asymmetric hydrogenation of an *N*-acyl enamine, where only the heterocombination of ligands gave almost complete enantioselectivity.

**Scheme 14:**
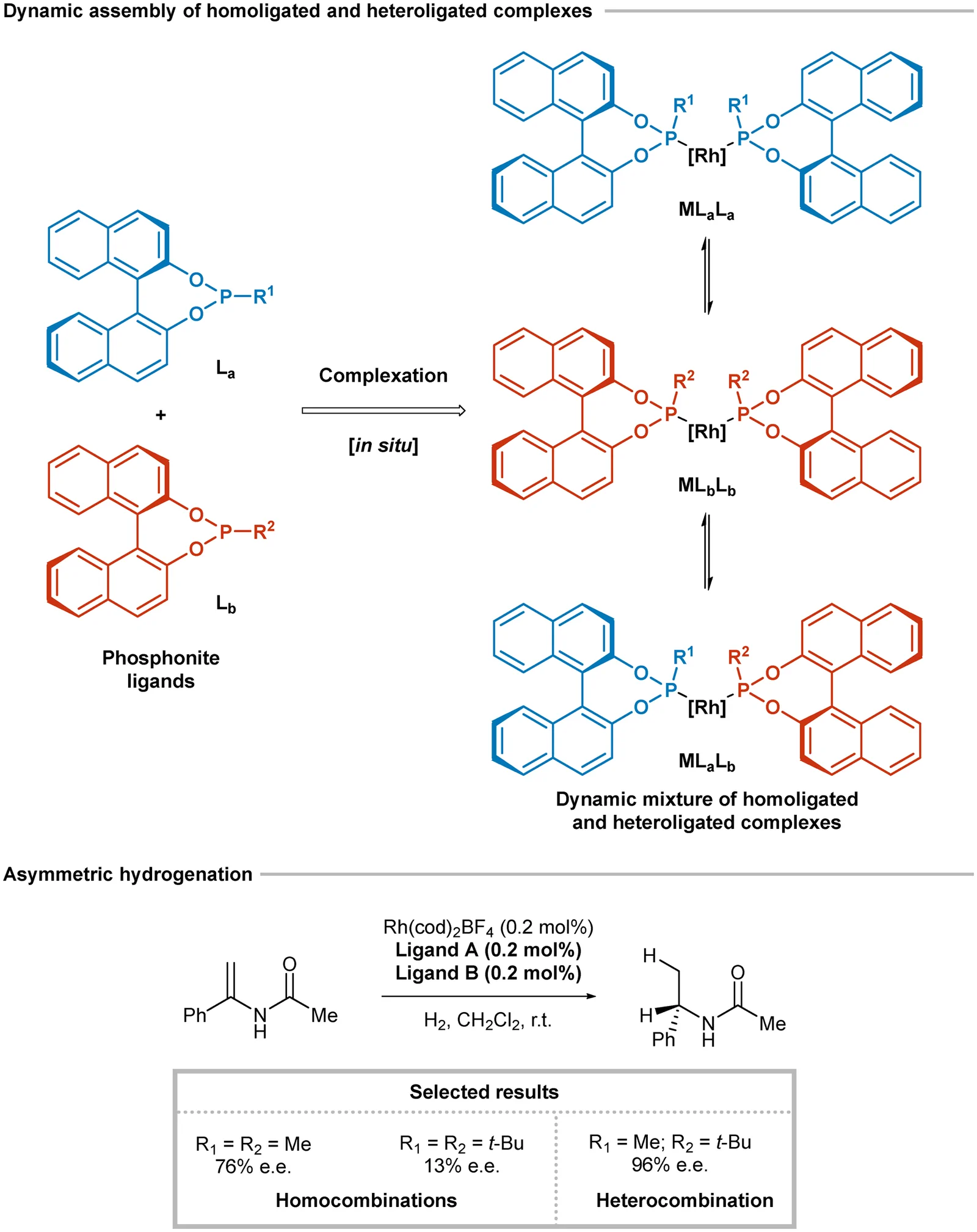
Dynamic formation of homoligated and heteroligated Rh–phosphite complexes and their application in enantioselective alkene hydrogenation as reported by Reetz and co-workers.^62^

One advantage that adopting dynamic combinatorial chemistry gives is the ability to simultaneously evaluate a mixture of candidates in parallel. This was demonstrated by the group of Ramström who used reversible imine formation to generate a library of bifunctional organocatalysts for the Morita–Baylis–Hillman (MBH) reaction (Scheme 15).^68^ Their two modules – one containing a hydrogen-bond donor and a primary amine (**Module 12A**) and the other a nucleophile and an aldehyde (**Module 12B**) – could be easily combined *in situ* by imine formation. Since this reaction is reversible, a thermodynamic mixture of 16 different catalysts is formed. Removal of water by adding molecular sieves locks the equilibrium mixture, which was then evaluated in the model reaction. To identify the optimal catalyst, a deconvolution approach was devised that relied on the selective replacement of one of their active modules with a module that does not include a hydrogen-bond donor or nucleophilic functionality. Thus, they identified that the combination of modules **12A-1** and **12B-2** gave the most effective catalyst.

**Scheme 15:**
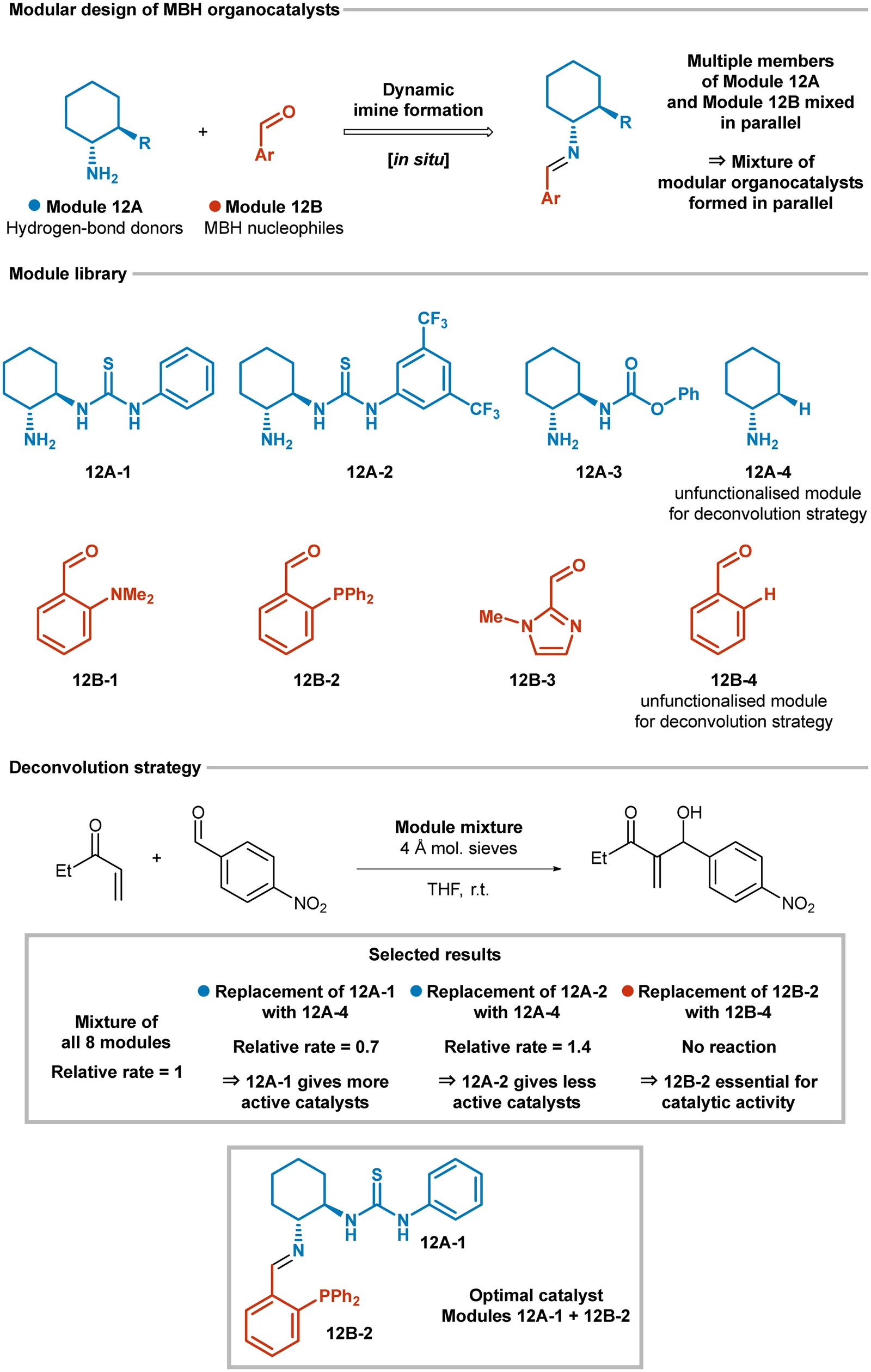
Dynamic formation of catalyst mixtures for the Morita–Baylis–Hillman reaction of as reported by Ramström and co-workers.^68^

## 6 Two-dimensional metal–ligand libraries

Another route to designing two-dimensional modular catalyst libraries is by using different metals as one module and different ligands as the other module. In 1996, Burgess and co-workers reported a seminal example of 2D metal–ligand screening (Scheme 16).^69^ Using high-throughput experimentation approaches, they evaluated a diverse library of chiral multidentate ligands (**Module 13A**) with various metal salts (**Module 13B**) for an intramolecular C–H insertion reaction. Solvent selection was also evaluated, thus giving a three-dimensional exploration of reaction space in their 96-well plate. The ligands and metal salts were complexed *in situ* and used in the catalysis without purification, providing a simple and rapid workflow. Although this screen did identify the same metal–ligand combination previously reported to be optimal, an improvement in yield and stereoselectivity was achieved by switching of the solvent. It also led to the unexpected discovery of Ag-complexes as effective catalysts for C–H insertion reactions.

**Scheme 16:**
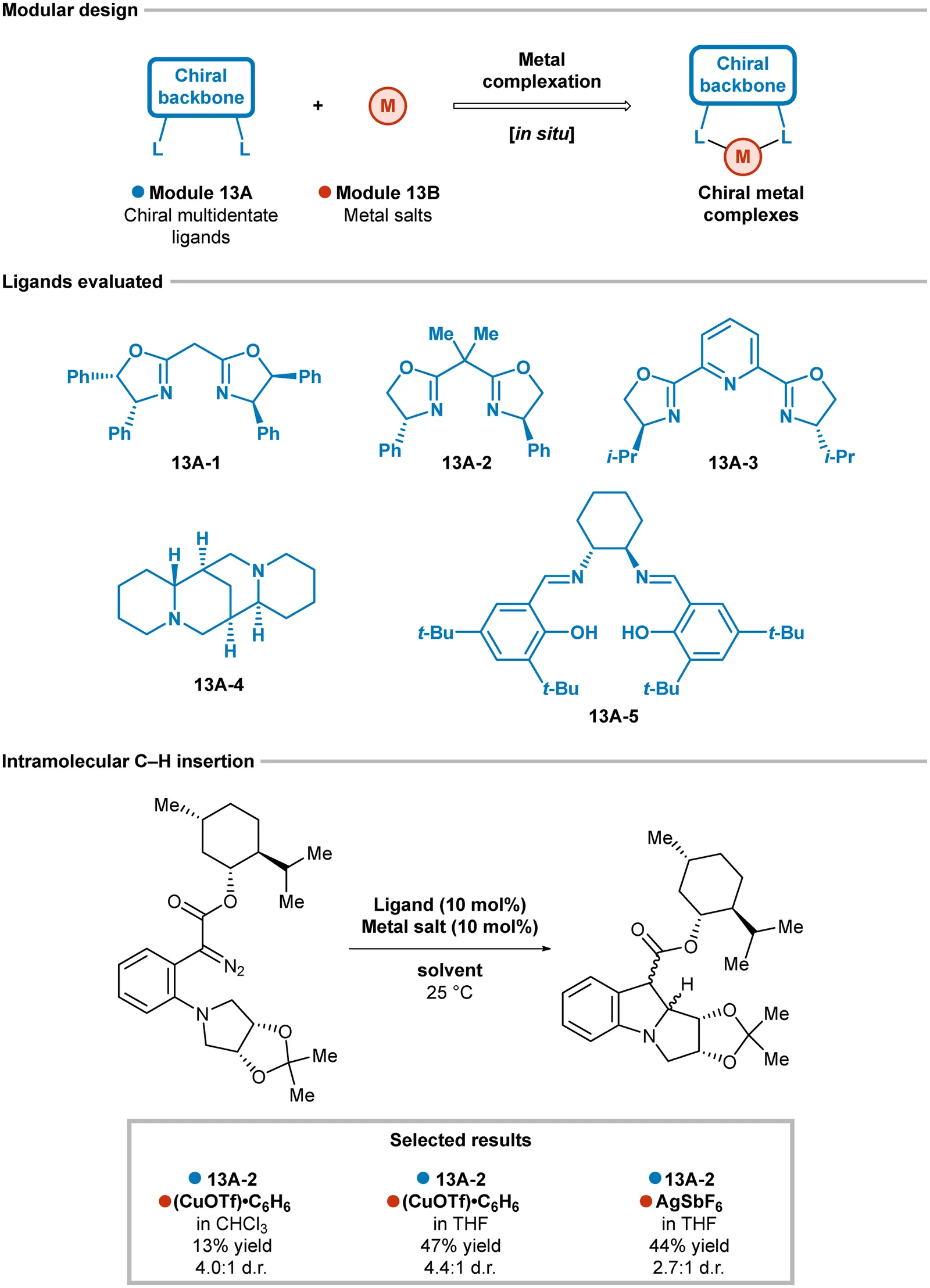
Design of a two-dimensional metal–ligand library for intramolecular C–H insertion as reported by Burgess and co-workers.^69^

Later, Bhawal and co-workers developed a workflow to generate a two-dimensional library of phosphine-Lewis acid complexes as ligands for Ni-catalysis (Scheme 17).^70^ This was motivated by the numerous examples of Ni-Lewis acid co-catalysis.^71,72^ Their strategy relied on the incorporation of a Brönsted acidic functionality into a triarylphosphine (**Module 14A**) that undergoes deprotonative metallation with an organometallic reagent (**Module 14B**) that acts as the source of the Lewis acidic cation. Depending on the valency of the metal ion and the organometallic species used, either monophosphine- or bisphosphine Lewis acid complexes were formed. Their study showed the monophosphine complex formed using KHMDS and the bisphosphine complex formed using (*n*-Bu)_2_Mg were the most effective. Further work demonstrated the benefits of incorporating the Lewis acidic cation.

**Scheme 17:**
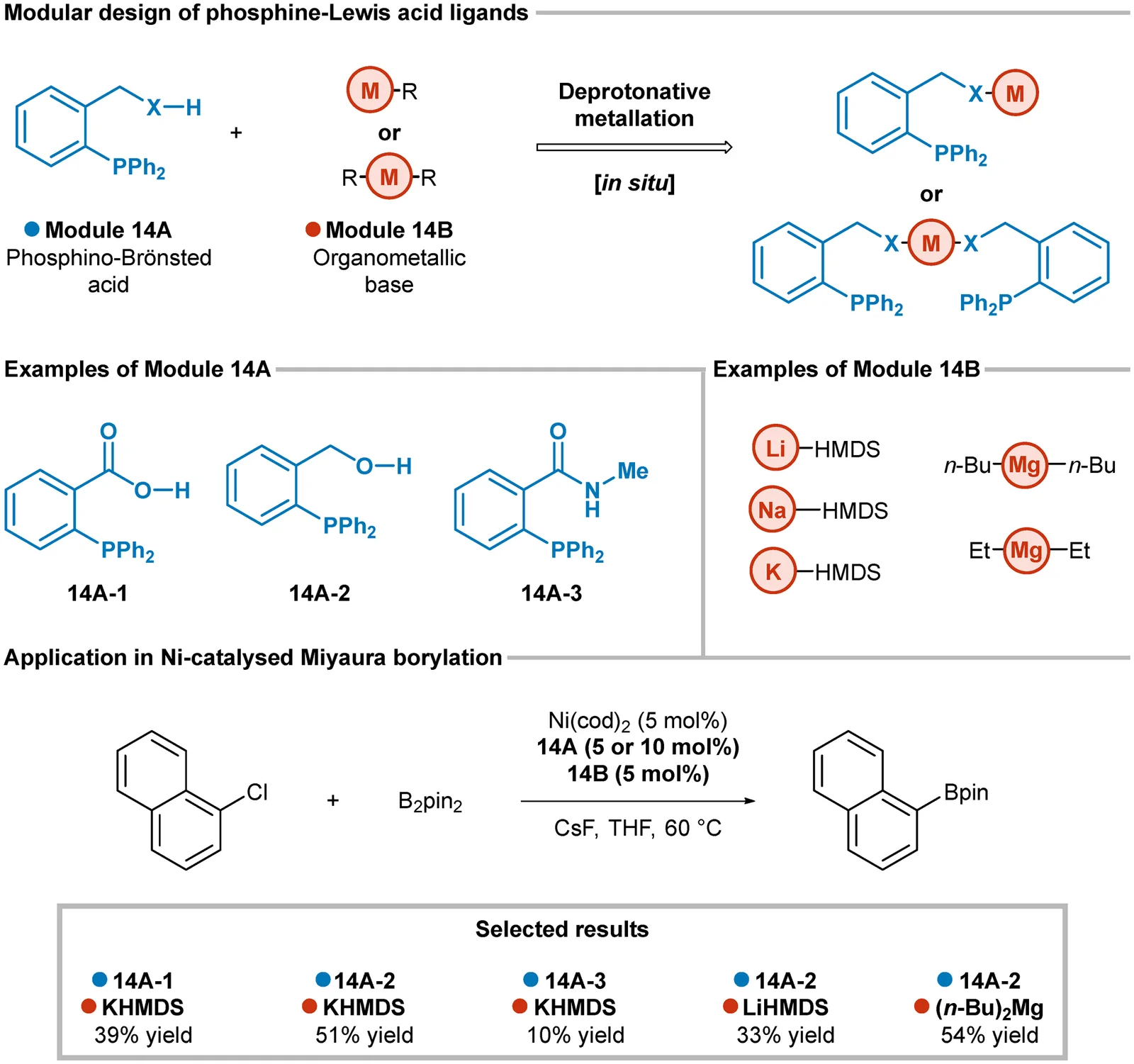
Design of modular phosphine-Lewis acid ligands for Ni-catalysis as reported by Bhawal and co-workers.^70^

## 7 Conclusions

In this perspective, we have discussed various approaches for implementing modular design strategies for catalyst discovery and optimisation. We have examined examples that have used a variety of strategies to assemble the active catalysts. Each of these approaches have their own distinct benefits and potential drawbacks.

Using covalent interactions to assemble the modular catalyst is conceptually the simplest approach to design and can make use of robust synthetic transformations typically using easily accessible functional groups. In most designs, though, the modular catalyst assembly must be performed *ex situ*, making this approach more laborious. A notable exception is the BIMP catalysts, pioneered by the Dixon group,^24^ which are accessed by the Staudinger reaction and as this only produces N_2_ as a by-product, enables catalyst assembly to be performed *in situ*.

By contrast, non-covalent interactions generally allow catalyst assembly to be performed *in situ*. While this avoids the need to synthesise each individual active catalyst, there are other challenges associated with this approach including the need for careful control of the non-covalent interactions to give the desired catalyst assembly. As a result, this approach is often reliant on more complex functional groups making it harder to access the requisite modules.

Dynamic catalyst assembly relies on reversible interactions, both covalent and non-covalent, to assemble the modular catalysts. To date, examples of this approach have used relatively easy to access modules and is reliant on *in situ* formation of the modular catalyst. Since this approach enables the interconversion of different catalyst species, it is typically performed to evaluate multiple catalysts in the same reaction vessel. Although this approach can potentially evaluate large catalyst libraries more rapidly, there are challenges, particularly in identifying the optimal catalyst. This can be further complicated as the exact concentrations of each catalyst within the mixture is often not known and can be sensitive to the reaction conditions.

Metal–ligand libraries offer a low-barrier pathway to implementing modular design for catalyst discovery. This generally works under the assumption that formation of the metal complexes proceeds smoothly and can be performed *in situ*. With the availability of many suitable pre-catalysts and ligands, this approach can be rapidly used but its drawbacks do include the potential sensitivity of some metal pre-catalysts and also overlooks the true structure of the metal complex formed, which may lead to misleading results.

Although this article has focussed on bimodular catalysts, we do not wish to detract from the utility of catalysts comprised of three or even more modules. The remarkable catalytic activity of enzymes, which can be viewed as a linear assembly of multiple modules, demonstrates the potential of catalysts assembled from even larger numbers of modules. Increasing the number of dimensions in the potential catalyst space leads to an exponential increase in the size and diversity of the catalyst library but raises challenges with regards to efficiently assembling the catalysts and also designing suitable evaluation strategies.^73^

The advent of new technologies, both with regards to automation and computation, offers to facilitate and further enhance the utility of adopting a modular catalyst design strategy. Advances in high-throughput experimentation have already been demonstrated by Burgess and co-workers to aid in the automated evaluation of modular catalyst libraries.^69^ As high-throughput experimentation is often used to perform 2-dimensional screening, this provides an ideal solution to evaluate bimodular catalysts and we anticipate increased access to this technology will facilitate the comprehensive evaluation of the large and diverse catalyst libraries afforded through using a modular catalyst design strategy.^74–77^ The development of novel catalyst evaluation approaches, as demonstrated by the groups of Breit,^48^ Ooi^57^ and Ramström^68^ can further accelerate the process of catalyst optimisation.

Consequently, this will be beneficial for machine learning which requires informative and carefully curated data sets. By evaluating modules that provide a diverse representation of the catalyst space under consideration, predictive models for further refining an idealised 3-dimensional arrangement of the catalyst species and substrates can be developed.^78,79^ The reporting of both productive and unproductive module combinations will be crucial for building these predictive models.

However, it is imperative to be prudent when implementing a modular catalyst design; having a large library of poorly designed modular catalysts will be costly to evaluate and offer limited meaningful insight.

In summary, this perspective article highlights the advances that have been made in catalysis through the use of modular catalyst design strategies. Judicious choice of transformations or non-covalent interactions to assemble the active catalyst is imperative in extracting the greatest value from this approach. The conceptualisation of new approaches to assemble modular catalysts and perform the evaluation of the catalyst libraries will further enhance its utility. We hope this article will encourage the uptake of modular design strategies and provide new opportunities for catalyst discovery and optimisation.

## Author contributions

F. M. contributed to writing – original draft, and writing – review & editing. B. S. G., E. M. D. and K. F. C. contributed to writing – review & editing. B. N. B. contributed to conceptualisation, writing – original draft, and writing – review & editing.

## Conflicts of interest

There are no conflicts to declare.

## Data Availability

No primary research results, software or code have been included, and no new data were generated or analysed as part of this Perspective article.
